# Leg length, sitting height and postmenopausal breast cancer risk

**DOI:** 10.1038/bjc.2012.244

**Published:** 2012-06-07

**Authors:** L Mellemkjær, J Christensen, K Frederiksen, J L Baker, A Olsen, T I A Sørensen, A Tjønneland

**Affiliations:** 1 Virus, Lifestyle and Genes, Danish Cancer Society Research Center, Strandboulevarden 49, DK-2100 Copenhagen, Denmark; 2 Institute of Preventive Medicine, Copenhagen University Hospitals, Øster Søgade 18,1. DK-1357 Copenhagen K, Denmark

**Keywords:** female breast cancer, adult height, leg length, sitting height, oestrogen receptor status, histology

## Abstract

**Background:**

Tallness has consistently been associated with an increased risk of breast cancer. We investigated the association further by decomposing height into leg length and sitting height.

**Methods:**

From the prospective Danish cohort ‘Diet, Cancer and Health’, 23 864 postmenopausal women enrolled during 1993–1997 were followed for a diagnosis of breast cancer in the Danish Cancer Registry through 2009.

**Results:**

The incidence rate ratios for breast cancer were 1.11 (95% CI=1.06–1.16) for each 5 cm increase in total height and 1.09 (95% CI=1.01–1.17) and 1.14 (95% CI=1.04–1.25) for each 5 cm increase in leg length and sitting height, respectively. There was no statistical significant difference between the associations for leg length and sitting height (*P*=0.47).

**Conclusion:**

Leg length does not seem to be more strongly associated with breast cancer among postmenopausal women than sitting height.

Adult height has consistently been found to be positively associated with breast cancer (van den Brandt *et al*, 2000; Gunnell *et al*, 2001; Lahmann *et al*, 2004; Green *et al*, 2011). The underlying cause for the association is unknown, but it has been suggested that factors associated with prepubertal growth are involved. Between birth and puberty, the legs grow relatively faster than other parts of the body (Fredriks *et al*, 2005), and leg length may therefore be a marker of prepubertal growth. If factors associated with prepubertal growth are underlying the association between adult height and breast cancer, it could be expected that the association between leg length and breast cancer would be stronger than that between sitting height and breast cancer. However, studies that have examined the two components of height in relation to risk of breast cancer have shown mixed results (Albanes *et al*, 1988; Freni *et al*, 1996; Swanson *et al*, 1996; Lawlor *et al*, 2003; Whitley *et al*, 2009). We investigated overall and subtype-specific risk of breast cancer according to leg length and sitting height in the largest cohort investigated so far including 23 864 postmenopausal women.

## Materials and methods

Between December 1993 and May 1997, all women without a prior cancer diagnosis aged 50–64 years born in Denmark and residents in the greater Copenhagen and Aarhus areas were invited to participate in the study ‘Diet, Cancer and Health’ a total of 29 875 accepted, corresponding to 37% of the women invited (Tjonneland and Overvad, 2000). The women attended a study clinic where they completed a questionnaire on reproductive factors, health-related issues as well as social and lifestyle factors. The study protocol was approved by the regional scientific ethics committees on human studies.

Women included in the present study were postmenopausal at baseline (defined as women who had not menstruated during the past 12 months (*N*=20 562) (including those who had undergone bilateral oophorectomy and/or hysterectomy) and women who had menstruated within the past 12 months and were current users of HRT (*N*=4125)).

Trained laboratory technicians obtained anthropometric measurements at the study clinics. Height was measured when the participant was standing without shoes, and sitting height was measured from the vertex of the head to the seated buttocks. Both measurements were recorded to the nearest 0.5 cm. Leg length was calculated as height minus sitting height, and relative leg length as the leg length divided by height. In all, 21 subjects were excluded because the height measurement was missing.

All study subjects were linked to the Central Population Register for information on vital status and emigration and to the Danish Cancer Registry (Gjerstorff, 2011) for information on cancer occurrence. For all breast cancer cases, information on histological subtype and oestrogen receptor (ER) status was obtained from the Danish Breast Cancer Cooperative Group Database (Moller *et al*, 2008). Study subjects were followed for breast cancer from age at visit to the study clinic (baseline) until age at diagnosis of breast cancer, age at diagnosis of other cancers (except non-melanoma skin cancer), age at death, emigration or December 31, 2008, whichever came first. In total, 2066 women were censored during follow-up because they were diagnosed with another cancer.

Incidence rate ratios (IRRs) were based on Cox regression models, with age as the time axis. Adjustments were made for parity (entered as two variables: the categorical variable parous/nulliparous and the continuous variable number of births), age at birth of first child (continuous), age at menarche (continuous), use of HRT (current/past/never), duration of HRT (continuous), alcohol intake (continuous), length of schooling (low: ⩽7 years/medium: 8–10 years/high: >10 years) and BMI at baseline (continuous). Women with missing information on these covariates (except age at menarche) were excluded from the analyses, thus, 23 864 women remained in the study cohort.

To investigate whether the positive association between adult height and breast cancer is primarily due to an association with leg length, we tested whether the associations with leg length and sitting height were similar in a model including leg length and sitting height as well as covariates (Wald test). We also calculated IRRs for ER-positive and ER-negative breast cancer as well as IRRs for ductal and lobular breast cancer, respectively (298 cases who had unknown ER status and 370 cases who had other or unknown histological subtypes were omitted from these analyses). A heterogeneity test was performed for IRRs by histological subtype and by ER status. Tests were based on the likelihood ratio test statistics calculated from Cox’s partial likelihood. Confidence intervals were based on Wald’s test of the corresponding regression parameters, that is, on the log scale for the IRRs. The SAS procedure PHREG was used for statistical analyses (SAS Institute, Cary, NC, USA).

## Results

A total of 1209 breast cancer cases were identified during a mean follow-up period of 11.8 years (range 0.0–15.1 years) among the 23 864 postmenopausal women. The IRR for breast cancer was 1.13 (95% CI=1.08–1.19) times higher for each 5 cm increment in height after adjustment for age (Table 1). There was no effect on the risk estimate of further adjustment for age at menarche (IRR=1.13; 95% CI=1.08–1.19), whereas adjustment for length of schooling had a minimal effect (IRR=1.12; 95% CI=1.07–1.18). Adjustment for the full list of potential covariates only changed the risk estimate slightly (IRR=1.11; 95% CI=1.06–1.16). The same pattern with little change in IRRs by these adjustments was seen for sitting height and leg length (data not shown). The fully and mutually adjusted IRRs were 1.14 (95% CI=1.04–1.25) for each 5 cm increments in sitting height and 1.09 (95% CI=1.01–1.17) for each 5 cm increments in leg length, respectively. The results were largely the same when weight was included as a covariate in the models instead of BMI. The associations with breast cancer for leg length and sitting height were not significantly different (*P*=0.47).

Height was positively associated with ER-positive breast cancer but not with ER-negative breast cancer, although there was no statistically significant heterogeneity in the risk estimates (Table 2). Positive associations were also seen for both ductal and lobular breast cancer, although due to small numbers the risk estimate for lobular breast cancer did not attain statistical significance.

## Discussion

In our large prospective study, we found that both components of height – leg length and sitting height were positively associated with risk of breast cancer among postmenopausal women. Height was positively associated with ER-positive breast cancer and with both ductal and lobular breast cancer.

Our study had the advantage of utilising data from a large prospective cohort that included standardised measurements of heights performed by a trained technician and information on a variety of potential confounders. Also, we had reliable follow-up and breast cancer data. However, we lacked information on childhood growth and birth size that potentially could help elucidate the underlying mechanisms for the association between height and breast cancer.

A positive association between adult height and breast cancer has been found in a large number of studies (van den Brandt *et al*, 2000; Gunnell *et al*, 2001; Lahmann *et al*, 2004; Green *et al*, 2011). The association often remains after adjustment for breast cancer risk factors, such as education and socioeconomic factors, as also observed in our data.

Adult height may represent a proxy for *in utero* and childhood growth. Birth weight and length (Silva I dos Santos *et al*, 2008) as well as early (Li *et al*, 2000; Li *et al*, 2007) and increased childhood growth (Hilakivi-Clarke *et al*, 2001; Ahlgren *et al*, 2004) have been linked to breast cancer risk. Nutritional status before puberty has been hypothesised to be important for development of breast cancer later in life, and some data have suggested that leg length is a more sensitive marker of nutritional influences during prepubertal growth than total height (Wadsworth *et al*, 2002). Our finding of a positive association with breast cancer for both leg length and sitting height is in accordance with findings from the cross-sectional British Women’s Heart and Health Study (Lawlor *et al*, 2003). Other studies have shown a positive association with childhood trunk length but not leg length (Whitley *et al*, 2009), no clear trend for neither leg length nor sitting height (Albanes *et al*, 1988) and no association with sitting height (Freni *et al*, 1996). Overall, these studies and our study do not support that leg length is more strongly associated with breast cancer risk than sitting height, thus suggesting that factors related to prepubertal growth are not predominantly underlying the association between height and breast cancer.

Previous studies have generally not shown any clear differences for overall height associations by ER/PR status of the breast cancer cases (Sellers *et al*, 2002; Colditz *et al*, 2004; Rosenberg *et al*, 2006; Borgquist *et al*, 2009; John *et al*, 2011) in accordance with our result of no significant difference. Studies investigating variations in risk by histological subtype of breast cancer have also found no significant heterogeneity across types (Li *et al*, 2003, 2006) in line with our results.

Our findings do not support that leg length is the component of height most strongly associated with breast cancer among postmenopausal women, thus decomposing height into leg length and sitting height did not add further information to explain why tall women are at greater risk of breast cancer than short women.

## Figures and Tables

**Table 1:** Percentiles for adult height, sitting height, leg length and relative leg length and IRR for breast cancer by these measurements among 23 864 postmenopausal women in the Diet, Cancer and Health cohort

	**Percentiles**						**Incidence rate ratios**					
	**Cohort (** ***n*** **=23 864)**			**Cases (** ***n*** **=1209)**			**Age-adjusted**		**Adjusted** a		**Mutually adjusted** a ^,^ b	
**Characteristic**	**5**	**50**	**95**	**5**	**50**	**95**	**IRR** c	**95% CI**	**IRR** c	**95% CI**	**IRR** c	**95% CI**
Height (m)	1.54	1.64	1.74	1.55	1.65	1.75	1.13	1.08–1.19	1.11	1.06–1.16		
Sitting height (m)	0.81	0.87	0.92	0.82	0.87	0.92	1.22	1.12–1.34	1.18	1.08–1.29	1.14	1.04–1.25
Leg length^d^ (m)	0.71	0.78	0.85	0.71	0.78	0.85	1.15	1.07–1.23	1.12	1.04–1.20	1.09	1.01–1.17
Relative leg length^e^	0.45	0.47	0.49	0.45	0.47	0.49	1.02	0.98–1.07	1.02	0.98–1.06		

Abbreviations: CI=confidence interval; IRR=incidence rate ratios.

a Adjusted for age, parity, age at birth of first child, age at menarche, use of HRT, duration of HRT, alcohol intake, length of schooling and BMI.

b Sitting height and leg length mutually adjusted.

c Per 5 cm increase except for relative leg length. Per 0.01 increase for relative leg length.

d Subischial leg length=height – sitting height.

e Relative leg length=subischial leg length/height.

**Table 2:** Incidence rate ratios for subgroups of breast cancer in relation to adult height, sitting height and leg length among 23 864 postmenopausal women in the Diet, Cancer and Health cohort

	**ER-positive breast cancer (** ***N*** **=742)**				**ER-negative breast cancer (** ***N*** **=169)**				
	**Age-adjusted**		**Adjusted** a		**Age-adjusted**		**Adjusted** a		
**Baseline measurements**	**IRR**	**95% CI**	**IRR**	**95% CI**	**IRR**	**95% CI**	**IRR**	**95% CI**	***P*** **-value** b
Height (per 5 cm)	1.10	1.04–1.17	1.08	1.01–1.15	1.02	0.90–1.16	1.00	0.88–1.14	0.31
Sitting height (per 5 cm)	1.16	1.03–1.30	1.10	0.98–1.24	0.93	0.74–1.18	0.89	0.70–1.14	0.12
Leg length^c^ (per 5 cm)	1.11	1.02–1.21	1.06	0.97–1.16	1.08	0.90–1.29	1.08	0.90–1.31	0.79
									
	**Ductal breast cancer (** ***N*** **=710)**				**Lobular breast cancer (** ***N*** **=129)**				
	**Age-adjusted**		**Adjusted** a		**Age-adjusted**		**Adjusted** a		
	**IRR**	**95% CI**	**IRR**	**95% CI**	**IRR**	**95% CI**	**IRR**	**95% CI**	***P*** **-value** d
Height (per 5 cm)	1.07	1.01–1.14	1.06	1.00–1.13	1.16	1.00–1.34	1.10	0.95–1.28	0.70
Sitting height (per 5 cm)	1.09	0.97–1.22	1.04	0.92–1.18	1.24	0.94–1.63	1.13	0.85–1.52	0.59
Leg length^c^ (per 5 cm)	1.10	1.01–1.20	1.07	0.98–1.18	1.19	0.97–1.46	1.08	0.87–1.35	0.90

Abbreviations: CI=confidence interval; ER=oestrogen receptor; IRR=incidence rate ratios.

a Adjusted for age, parity, age at birth of first child, age at menarche, use of HRT, duration of HRT, alcohol intake, length of schooling and BMI. Sitting height and leg length also mutually adjusted.

b *P*-value for test for heterogeneity between adjusted IRRs for ER-positive and ER-negative breast cancer.

c Subischial leg length=height – sitting height.

d *P*-value for test for heterogeneity between adjusted IRRs for ductal and lobular breast cancer.
